# A vein-viewing application enabled detecting abdominal wall varices related to the presence of non-treated gastroesophageal varices: a cross-sectional study

**DOI:** 10.1186/s12880-021-00655-8

**Published:** 2021-08-09

**Authors:** Yoshiki Hoshino, Takaaki Sugihara, Suguru Ikeda, Yukako Matsuki, Takakazu Nagahara, Jun-ichi Okano, Hajime Isomoto

**Affiliations:** Division of Medicine and Clinical Science, Department of Gastroenterology and Nephrology, Faculty of Medicine, Tottori University, 36-1 Nishi-cho, Yonago, 683-8504 Japan

**Keywords:** Abdominal wall varices, Cirrhosis, Gastroesophageal varices

## Abstract

**Background:**

Gastroesophageal varices (GOV) are a life-threatening complication in chronic liver disease. A method for non-invasively predicting GOV is crucial for management. This study aimed to determine whether a vein-viewing application can detect abdominal wall varices (AWV) and elucidate the relationship between AWV and GOV.

**Methods:**

One-hundred patients with chronic liver diseases were prospectively enrolled. All the patients underwent esophagogastroduodenoscopy within three months of the enrollment. Unmanipulated images (UI) and vein-weighted images (VWI) were taken for assessing AWV by a vein-viewing application on iPhone. Two doctors independently evaluated both image types. We defined the grading of both UI and AWV as grade 0 (non-detectable), grade 1 (slightly detectable), and grade 2 (distinct).

**Results:**

The causes of liver diseases among the 71 men and 29 women (median age, 70.5 yr) included Hepatitis B (n = 19), Hepatitis C (n = 21), alcoholism (n = 33), primary biliary cholangitis (n = 3), autoimmune hepatitis (n = 4) and others (n = 20). GOV was indicated in ﻿60﻿ patients, and half of them had not been treated previously (non-treated). VWI could significantly visualize AWV than UI (72% vs. 24%, p = 0.0005). The presence of cirrhosis (chronic hepatitis vs. cirrhosis = 64.6% vs. 91.4%, p = 0.004) and GOV (52.3% vs. 74.3%, p = 0.032) were significantly higher in the VWI-AWV grade 2 group. Multivariate analysis demonstrated that VWI-AWV grade 2 was an independent factor related to the presence of non-treated GOV [OR = 3.05 (1.24–7.53), p = 0.016].

**Conclusions:**

The vein-viewing application non-invasively detected AWV related to the presence of cirrhosis and GOV, and VWI-AWV grade 2 was an independent factor related to the presence of non-treated GOV.

## Background

Gastroesophageal varices (GOV) are present in about half of the patients with cirrhosis [1]. Variceal bleeding is a life-threatening complication which accounts for 10–30% of all upper gastrointestinal bleeding [2]. Esophagogastroduodenoscopy (EGD) is the gold standard for the detection of GOV. Disadvantages of endoscopy include the risk of sedation, higher cost, bleeding, and risk of aspiration [3]. However, no recommendations on screening of GOV has been made in Japan [4]. Many less invasive methods for screening of GOV have been investigated [5]. Serum biomarkers including platelet count, FIB-4 index, aspartate aminotransferase to platelet ratio index (APRI), liver stiffness (LS), spleen stiffness (SS), LS-spleen diameter to platelet ratio, and Liver stiffness × spleen size/platelet count (LSPS) are reportedly useful for predicting esophageal varices [6–15]. The updated Baveno VI guidelines recommend that screening EGD can be avoided in patients with compensated advanced chronic liver disease who have liver stiffness < 20 kPa and a platelet count > 150,000/mm^3^ [16].

We focused on abdominal wall varices (AWV) for predicting GOV. Several prominent collateral veins radiating from the umbilicus are termed the caput-medusae. The caput-medusae sign is an indicator of portal hypertension. It describes engorged paraumbilical veins radiating from the umbilicus within the adipose tissue of the anterior abdominal wall, creating portosystemic anastomoses [17]. However, in clinical settings, it could not be commonly identified. It is now considered a rare finding. The use of infrared photography for the visualization of AWV is reported in the literature [18]. There are no specific modalities for visualizing AWV. Therefore, we used a vein-viewing application on the iPhone instead of an infrared camera. This can visualize the high-contrast image of the vein by boosting oxyhemoglobin/deoxyhemoglobin absorption contrast and reducing the contribution of superficially scattered and specularly reflected light to the overall image.

In this study, we aimed to evaluate the efficacy of the vein-viewing application for detecting the AWV in patients with chronic liver disease and elucidating the relationship between AWV and GOV.

## Methods

**Fig. 1:**
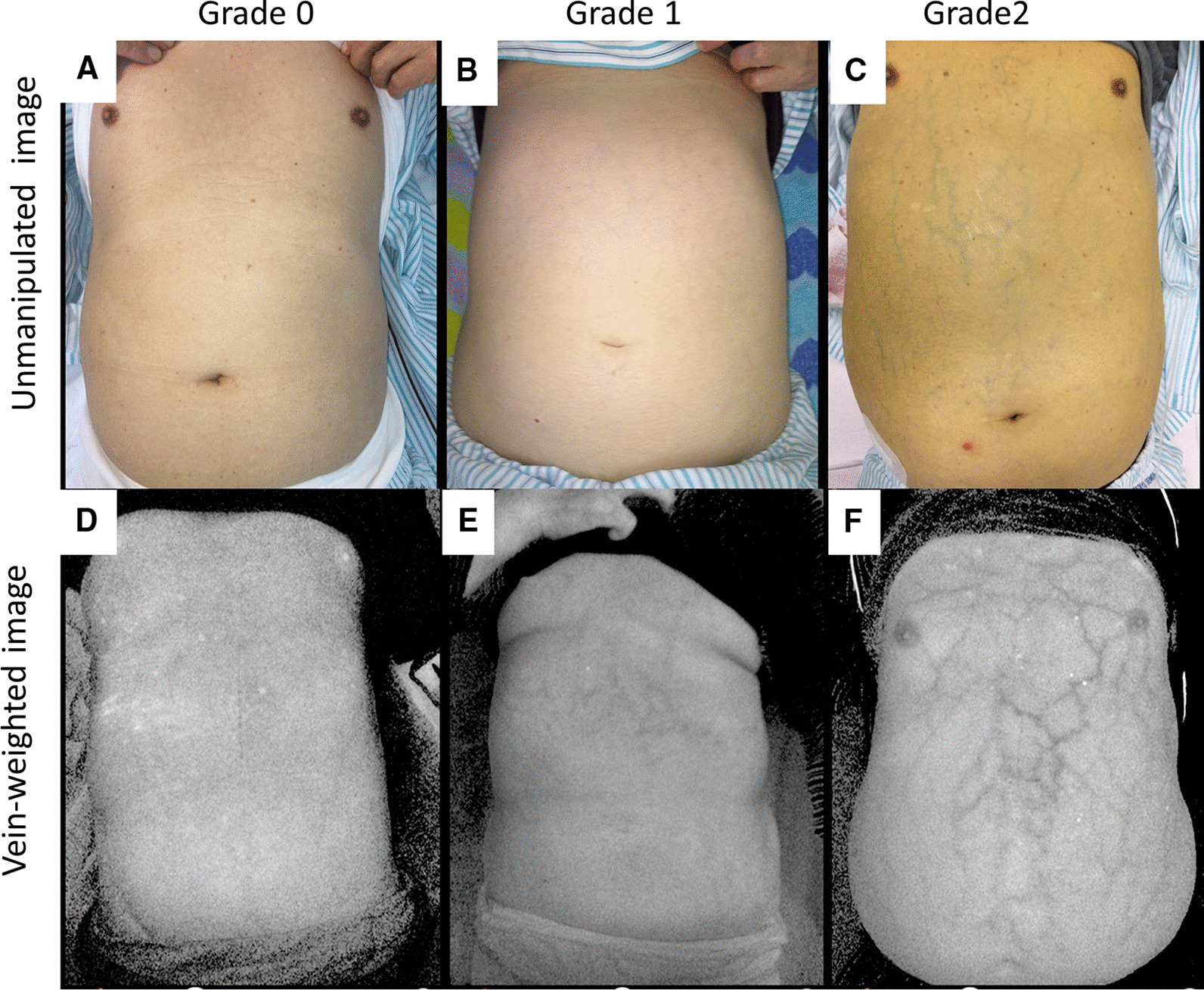
Classification of abdominal wall varices. **a** grade0; UI-non-detectable, **b** grade1; UI-slightly detectable, **c** grade2; UI-distinct, **d** grade0; VWI-non-detectable, **e** grade1; VWI-slightly detectable, **f** grade2; VWI-distinct, *UI* Unmanipulated image, *VWI* Vein-weighted image

This was a single-center, prospective, cross-sectional study. Between November 2018 and September 2020, one-hundred adult patients in our hospital with any chronic liver disease (including cirrhosis) were prospectively enrolled. All the patients underwent EGD within three months of inclusion. Patients with skin diseases of the abdominal wall were not enrolled because of skin discoloration preventing successful imaging. We obtained both unmanipulated images (UI) and vein-weighted images (VWI) with VeinSeek Pro (VeinSeek LLC, Los Angeles, CA) (https://www.veinseek.com/) for each patient. VeinSeek Pro for iPhone can be downloaded via App Store for iPhone (https://apps.apple.com/us/app/veinseek-pro/id1174536386). VeinSeek version 2 for android is also available; however, it does not work as well as VeinSeek Pro. We defined the grading of AWV as grade 0 (non-detectable), grade 1 (slightly detectable), and grade 2 (distinct) for both unmanipulated and VWI, respectively (Fig. 1). Both images were evaluated by two doctors (Dr. S and N) independently. We obtained the patient's information on biological gender, age, body mass index (BMI), and mental status (regarding hepatic encephalopathy) at the time of imaging. The following data: hemoglobin, total bilirubin, albumin, prothrombin time (PT), fibrosis index based on the four factors (FIB-4) index using age, aspartate transaminase (AST), alanine transaminase (ALT), and platelet values [19], and AST to platelet ratio index (APRI) [20] were also collected. The severity of cirrhosis was determined according to the Child–Pugh scoring system based on PT, albumin, bilirubin values, and the presence of encephalopathy or ascites. Patients were classified into Child A (5–6 points), B (7–9 points), and C (10–15 points) groups. Classification of GOV was according to the "general rules for recording the endoscopic findings of esophagogastric varices in Japan" [21]. Moreover, gastric varices were classified according to Sarin's classification [22]. Other abdominal imaging techniques (ultrasound, computerized tomography, or magnetic resonance imaging) were also applied for evaluating ascites. FibroScan measures of liver stiffness were also performed on patients without ascites.

### Statistical analysis

The Student's t-test and chi-square test were applied for comparing the two groups as defined by the cutoff criteria. One-way ANOVA was applied for multiple comparisons. Interrater reliability was assessed by the Cohen's kappa coefficient. A Kappa > 0.7 indicates agreement between two operators. Logistic regression analysis was applied for multivariate analysis. The Spearman rank-order correlation coefficient (shown as *rS*) was used for evaluating the correlation between two variables. All statistical tests were performed using StatFlex (Windows ver. 6.0; Artech, Osaka, Japan). Values are expressed as median (range) or mean with a standard error of the mean (SEM). Categorical variables are shown as numbers. Statistical significance was set at *p* < 0.05.

### Ethics approval and consent to participate

The study protocol was approved by the Institutional Review Board of Tottori University (No.18A152) under the guidelines of the 1975 Declaration of Helsinki. Written informed consent was obtained from all the participants.

## Results

### Baseline characteristics of the patients

The baseline characteristics of the patients are presented in Table 1. One-hundred patients [71 men, 29 women, median age, 70.5 (range, 20–87) years] were enrolled in this study. Their liver diseases were induced by the hepatitis B virus (HBV) (n = 19), hepatitis C virus (HCV) (n = 21), alcohol (n = 33), primary biliary cholangitis (PBC) (n = 3), autoimmune hepatitis (AIH) (n = 4), and others (e.g. Budd-Chiari syndrome and cryptogenic) (n = 20). The status of the underlying liver disease was chronic hepatitis in 26 patients and cirrhosis in 74 patients. Cirrhotic patients were classified into Child–Pugh class A (n = 37), B (n = 25), and C (n = 12), respectively. Esophageal varices were detected in 57 of the 100 patients examined and classified into F1 (n = 26), F2 (n = 29), and F3 (n = 2), respectively. Gastric varices were detected in 24 patients, and the form was classified into F1 (n = 14), F2 (n = 7), F3 (n = 3), respectively. According to Sarin’s classification, gastric varices were classified as GOV1 (n = 12), GOV2 (n = 8), GIV1 (n = 4), and GIV2 (n = 0), respectively. Thirty patients were treated for GOV before enrollment in the study by endoscopic variceal ligation (EVL, n = 21), endoscopic injection sclerotherapy (EIS, n = 6), balloon retrograde transvenous obliteration (B-RTO, n = 2), and Hassab's operation (n = 1), respectively. Therefore, GOV had been disappeared in three patients at enrollment. Among the 60 patients with GOV at enrollment, 33 patients had never been treated previously (non-treated group). Portal hypertensive gastropathy (PHG) was identified in 27 patients. Thirty-two patients had ascites. Fifty-nine patients had hepatocellular carcinoma (TNM stage I:II:III:IV = 14:28:19:5). Encephalopathy was diagnosed in only four patients. Splenomegaly was found in 53 patients. Portosystemic shunts (splenorenal, gastrorenal, and inferior mesenteric caval shunt-internal iliac vein) were found in 15 patients.

**Table 1:** Characteristics of patients

Patients	n = 100
Sex (male:female)	71:29
Age (years)	70.5 (20–87)
Etiology of liver disease	
HBV infection	19
HCV infection	21
Alcoholism	33
PBC	3
AIH	4
Others^†^	20
The status of the underlying liver disease	
Chronic hepatitis	26
Cirrhosis	74
Child–Pugh classification	
A:B:C	37:25:12
Esophageal varices^‡^	57
Location (Li:Lm:Ls)	10:34:13
Form (F1:F2:F3)	26:29:2
Color (Cw:Cb)	56:1
RC0:RC1:RC2:RC3	30:18:6:1
Gastric varices^†^	24
Location (Lg-c:Lg-f:Lg-cf)	13:10:1
Form (F1:F2:F3)	14:7:3
Color (Cw:Cb)	14:10
RC0:RC1:RC2:RC3	24:0:0:0
Sarin’s classification	
GOV1:GOV2:GIV1:GIV2	12:8:4:0
Past treatment of GOV	30
EVL:EIS:B-RTO:Hassab	21:6:2:1
Past rupture history	12
Portal hypertensive gastropathy	27
Encephalopathy	4
Ascites	32
Splenomegaly	53
Portosystemic shunt	15
SR:GR:IMC	8:6:1

*AIH* autoimmune hepatitis, *Cw* white varices, *Cb* blue varices, *F1* straight, small-caliber varices, *F2* moderately enlarged, beady varices, *F3* markedly enlarged, nodular or tumor-shaped varices, *GOV* gastroesophageal varices, *HBV* hepatitis B virus, *HCV* hepatitis C virus, *Ls* locus superior, *Lm* locus medialis, *Li* locus inferior, *Lg-c* adjacent to the cardiac orifice, *Lg-cf* extension from the cardiac orifice to the fornix, *PBC* primary biliary cholangitis, *RC* red color sign, *SR* splenorenal shunt, *GR* gastrorenal shunt, *IMC* inferior mesenteric caval shunt, Data are expressed as median (range)

^†^Including Budd–Chiari syndrome and cryptogenic, ^‡^including treated patients

### Abdominal wall varices visualization and classification

**Fig. 2:**
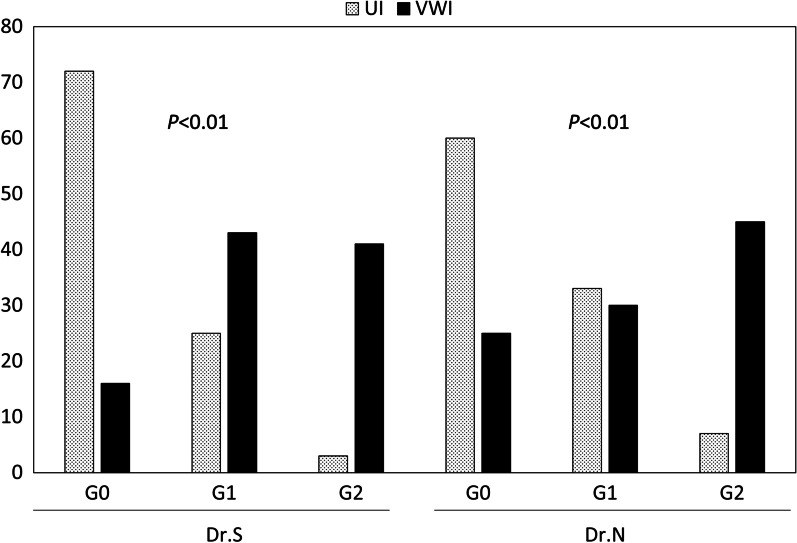
Classification of abdominal wall varices by the two doctors. Grade 0 was decreased in VWI, and grade 2 was increased in VWI in both doctors. *UI* Unmanipulated image, *VWI* Vein-weighted image

In UI, AWV was classified by the two doctors into grade 0 (n = 72, 59), grade 1 (n = 25, 33), and grade 2 (n = 3, 7), respectively. The kappa was 0.5. In VWI, AWV was classified by the two doctors into grade 0 (n = 16, 25), grade 1 (n = 43, 30), and grade 2 (n = 41, 45), respectively. Comparing UI and VWI, the AWV-positive cases (grade1 and 2) were significantly higher in VWI than UI (72% vs. 24%, *p* = 0.0005) (Table 2). In VWI, Grade 0 was decreased, and grade 2 was increased significantly in both doctors compared to the UI grading (*p* < 0.01) (Fig. 2). In VWI, the kappa was 0.55 for all grades; however, it was 0.72 for grade 2 classification. This finding implies that grade 2 judgment is more stable than other grades.

**Table 2:** Comparison between UI and VWI for depicting AWV

	VWI-negative	VWI-positive	Total
UI-negative	28	48	76
UI-positive	0	24	24^†^
Total	28	72^†^	100

UI-positive and VWI-positive cases are classified as grade1 and 2 by the two doctors

*UI* unmanipulated image, *VWI* vein-weighed image, *AWV* abdominal wall varices

^†^ AWV-positive cases were significantly higher in VWI than UI

### Comparing factors between negative and positive GOV

**Fig. 3:**
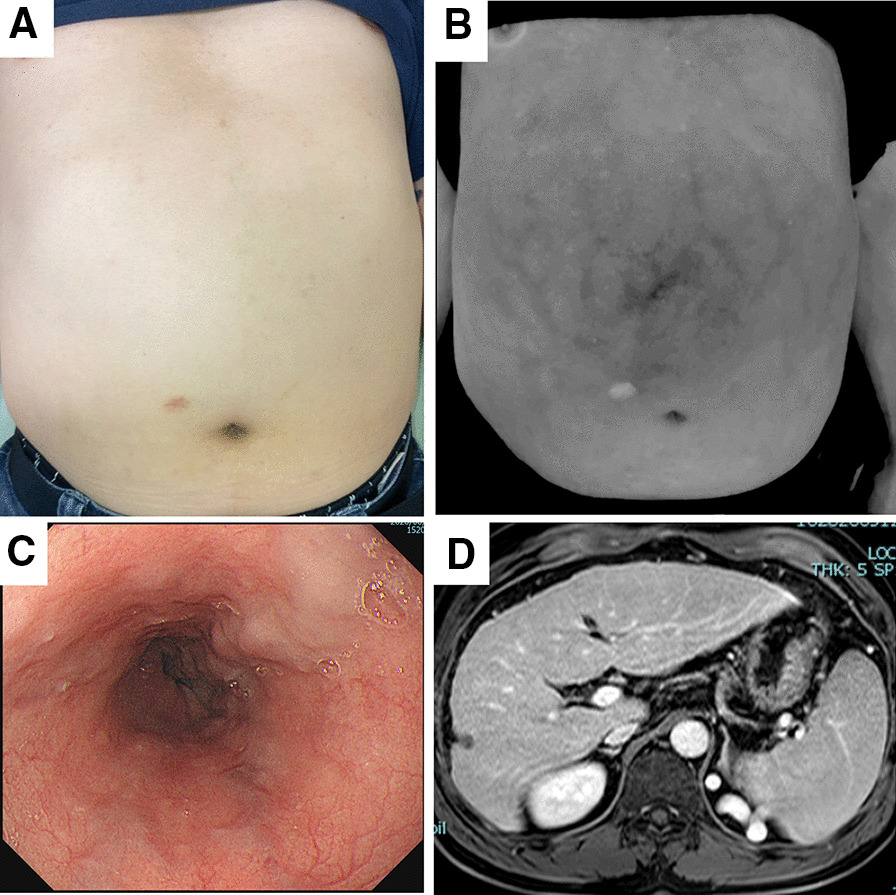
A representative case of VWI-AWV grade 2 whose UI-AWV was grade 0. **a** UI-AWV image classified grade 0, **b** VWI-AWV image classified grade 2, **c** Esophageal varices classified F2, **d** MRI showed cirrhosis and splenomegaly. *AWV* abdominal wall varices, *MRI* magnetic resonance of imaging, *UI* Unmanipulated image, *VWI* Vein-weighted image

The comparison factors between negative and positive GOV demonstrated that the presence of cirrhosis was higher in the positive GOV group (42.5% vs. 95%, *p* < 0.001). The presence of GOV was significantly higher in patients classified as VWI-AWV grade 2 by both doctors (22.5% vs. 43.3%, *p* = 0.032). For non-treated GOV (n = 70), the presence of GOV was also significantly higher in the patients classified as VWI-AWV grade 2 by both doctors (27.1% vs. 53.3%, *p* = 0.012). VWI could also detect grade 2 AWV in eight patients with no varices on their abdomen in UI (Fig. 3). In these eight patients, five patients (63%) had F2 esophageal varices and RC1 in two. Splenomegaly (35% vs. 65%, *p* = 0.003), VWI-AWV grade 2 (22.5% vs. 43.3%, *p* = 0.032), FIB-4 index (3.8 ± 2.5 vs. 6.1 ± 4.2, *p* = 0.003), APRI (1.0 ± 1.0 vs. 1.6 ± 1.2, *p* = 0.010), and liver stiffness (17.0 ± 16.7 vs. 28.0 ± 17.5 kPa, *p* = 0.011) were significantly higher in the positive group. In contrast, platelet count (151.7 ± 58.5 × 10^3^ vs. 107.7 ± 49.7 × 10^3^/mm^3^, *p* < 0.001), albumin (3.9 ± 0.7 vs. 3.6 ± 0.6 g/dL, *p* = 0.037), and PT (84.7 ± 23.0 vs. 73.3 ± 22.2%, *p* = 0.017) were significantly lower in the positive group (Table 3).

**Table 3:** Comparison of factors for prediction between patients with or without gastroesophageal varices

	Gastroesophageal varices^†^		*p* value
	Negative (n = 40)	Positive (n = 60)	
Gender (male/female)	27/13	44/16	0.529
Age (years)	72 (50–87)	69 (20–87)	0.050
Child–Pugh scores	7.2 ± 2.3	7.1 ± 2.2	0.885
Splenomegaly (yes/no)	14/26	39/21	0.003
UI-AWV (grade0/grade ≥ 1^‡^)	33/7	43/17	0.214
UI-AWV (grade 0–1/grade 2^‡^)	40/0	57/3	0.273
VWI-AWV (grade0/grade ≥ 1^‡^)	14/26	14/46	0.203
VWI-AWV (grade 0–1/grade 2^‡^)	31/9	34/26	0.032
Platelet count (10^3^/mm^3^)	151.7 ± 58.5	107.7 ± 49.7	< 0.001
FIB4 index	3.8 ± 2.5	6.1 ± 4.2	0.003
APRI	1.0 ± 1.0	1.6 ± 1.2	0.010
Liver stiffness^§^ (kPa)	17.0 ± 16.7	28.0 ± 17.5	0.011

Data are expressed as median (range) or mean ± SD

*APRI* aspartate aminotransferase to platelet ratio, *AWI* abdominal wall varices, *FIB-4*, fibrosis index based on the four factors, *UI* unmanipulated image, *VWI* vein-weighed image, *PT* percent prothrombin time

^†^Including treated patients, ^‡^classified by the two doctors, ^§^Liver stiffness was evaluated in 68 patients without ascites

### Comparing factors between grade 2 AWV and the others of VWI

The comparison of factors between VWI-AWV grade 2 and the other grade groups demonstrated that the presence of cirrhosis (CH vs. LC = 3/32, *p* = 0.004), GOV (52.3% vs. 74.3%, *p* = 0.032), ascites (24.6% vs. 47.1%, *p* = 0.023), and PHG (20% vs. 40%, *p* = 0.023) were significantly higher in the VWI-AWV grade 2 group. In contrast, platelet count (133.7 ± 60.2 × 10^3^ vs. 109.7 ± 48.9 × 10^3^/mm^3^, *p* = 0.046) was significantly lower in the VWI-AWV grade 2 group (Table 4).

**Table 4:** Comparison of factors between patients with VWI-AEV grade 2 and the others

	VWI-AWV		*p* value
	Grade 0–1 (n = 65)	Grade 2^†^ (n = 35)	
Gender (male/female)	45/20	26/9	0.595
Age (years)	71 (46–87)	69 (20–87)	0.290
chronic hepatitis/cirrhosis	23/42	3/32	0.004
Child–Pugh scores	6.25 ± 21.7	7.5 ± 2.4	0.020
Ascites (yes/no)	16/49	17/18	0.015
Splenomegaly (yes/no)	32/33^‡^	21/14	0.303
GOV (yes/no)	34/31	26/9	0.032
GOV > F2 (yes/no)	19/46	14/21	0.170
GOV > RC1 (yes/no)	13/52	12/23	0.116
Past treatment of GOV (yes/no)	17/48	13/22	0.253
Past rupture history (yes/no)	5/60	7/28	0.071
PHG (yes/no)	13/52	14/21	0.036
Platelet count (10^3^/mm^3^)	133.7 ± 60.2	109.7 ± 48.9	0.046
AST (U/L)	35.2 ± 17.6	46.0 ± 27.6	0.019
ALT (U/L)	27.9 ± 14.5	33.2 ± 18.4	0.118
Albumin (g/dL)	3.8 ± 0.7	3.5 ± 0.6	0.053
Total bilirubin (mg/dL)	1.1 ± 0.7	2.2 ± 4.5	0.050
PT (%)	80.0 ± 21.0	73.4 ± 26.1	0.180
FIB4 index	4.9 ± 4.1	5.7 ± 3.2	0.302
APRI	1.3 ± 1.2	1.6 ± 1.0	0.129
Liver stiffness^§^ (kPa)	23.1 ± 18.1	24.0 ± 18.0	0.849

Data are expressed as median (range) or mean ± SD

*AST* aspartate aminotransferase, *ALT* alanine aminotransferase, *APRI* aspartate aminotransferase to platelet ratio, *F2* moderately enlarged, beady varices, *FIB4* fibrosis index based on the four factors, *GOVs* Gastroesophageal varices, *PHG* portal hypertensive gastropathy, *PT* percent prothrombin time, *RC* red color sign

^†^Classified by the two doctors, ^‡^two patients had been undergone splenectomy, ^§^liver stiffness was evaluated in 68 patients without ascites

### Multivariate analysis of predicting factors for GOV

Multivariate analysis was applied for the factors related to GOV in Table 3. APRI and FIB-4 index were not selected because of including platelet count. Liver stiffness was also not selected because of the lack of data in 32 patients with ascites. Age ≥ 71 years [OR = 0.35 (0.14–0.85), *p* = 0.021] was an independent factor, and VWI-AWV grade2 [OR = 2.40 (0.91–6.33), *p* = 0.076] approached the borderline of significance. In this study, liver cirrhosis was lower in patients ≥ 71 years old (64% vs. 84%, *p* = 0.023). Therefore, age was negatively related to GOV (Table 5).

**Table 5:** Multivariate analysis of predicting factors for GOV

Factors		Multivariate analysis		
		Odds ratio	95%CI	*p*-value
Age	≥ 71 years^†^	0.35	0.14–0.85	0.021
Splenomegaly		2.52	0.73–8.69	0.144
Platelet count	< 80,000/mm^3‡^	2.26	0.85–6.02	0.102
VWI-AWV	Grade 2*	2.40	0.91–6.33	0.076

*APRI* aspartate aminotransferase to platelet ratio, *AWI* abdominal wall varices, *FIB-4* fibrosis index based on the four factors, *GOV* Gastroesophageal varices, *VWI* vein-weighed image

^†^According to the median age of all the patients, ^‡^according to the report by Burton et al. [23], *Classified by the two doctors

### Multivariate analysis of factors for non-treated GOV

Multivariate analysis was also applied for non-treated GOV. It was also applied both with and without liver stiffness. Only VWI-AWV grade2 was an independent factor related to non-treated GOV [OR = 3.05 (1.24–7.53), *p* = 0.016] (Table 6).

**Table 6:** Multivariate analysis of predicting factors for non-treated GOV

Factors		Multivariate analysis		
		Odds ratio	95%CI	*p* value
Age	≥ 71 years^†^	0.63	0.25–1.57	0.323
Platelet count	< 80,000/mm^3‡^	1.83	0.58–5.72	0.302
Splenomegaly		0.82	0.28–2.35	0.706
VWI-AWV	Grade 2*	3.05	1.24–7.53	0.016

*APRI* aspartate aminotransferase to platelet ratio, *AWI* abdominal wall varices, *FIB-4* fibrosis index based on the four factors, *GOV* Gastroesophageal varices, *VWI* vein-weighed image

^†^According to the median age of all the patients, ^‡^according to the report by Burton et al. [23], *Classified by the two doctors

### Relationship between parameters or shunts and VWI grading

**Fig. 4:**
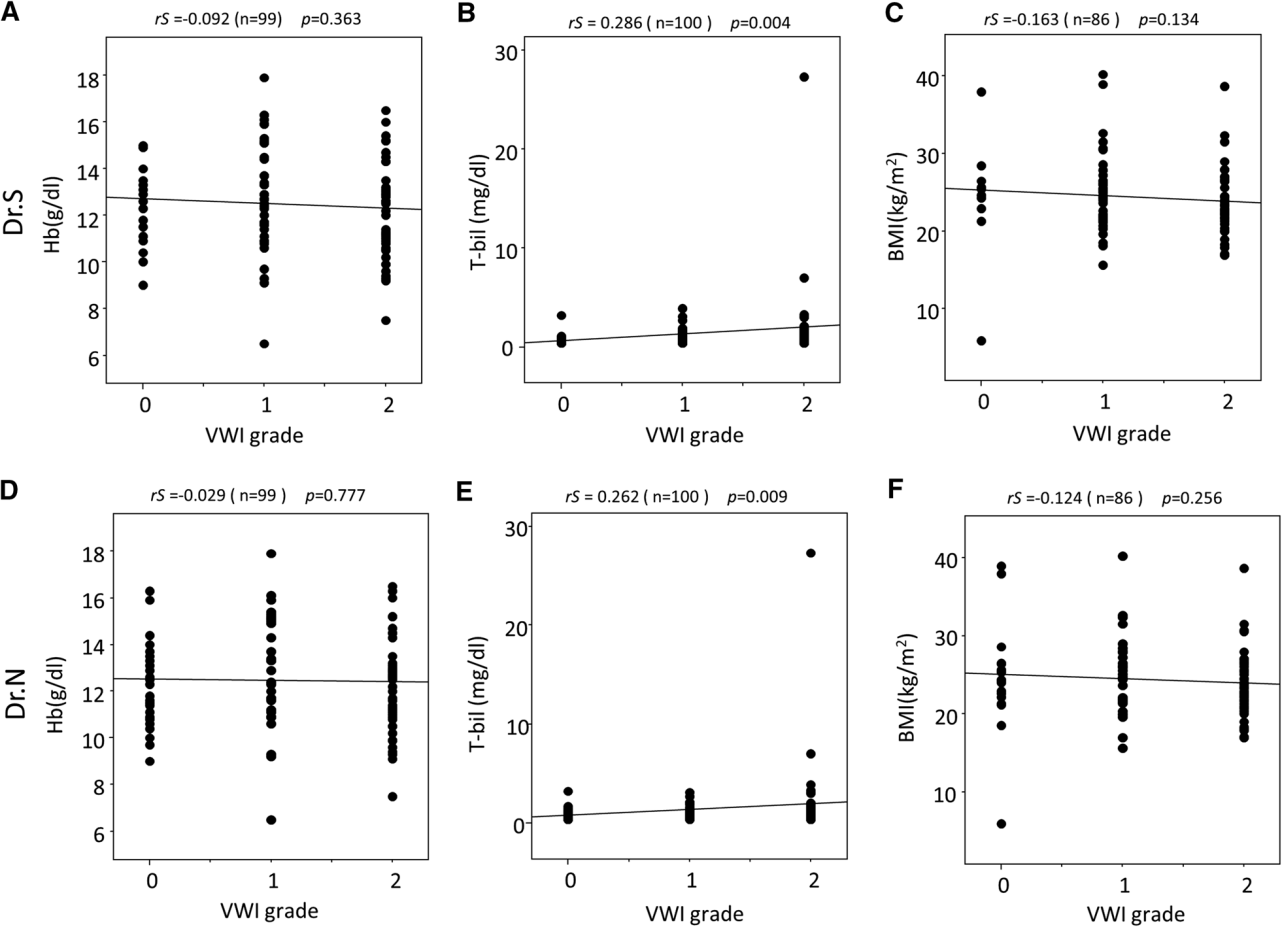
Correlation between parameters and VWI grading. **a** no correlation between Hb and VWI grade, **b** slight positive correlation between T-bil and VWI grade, and **c** no correlation between BMI and VWI grade by Dr.S. **d** no correlation between Hb and VWI grade, **e** slight positive correlation between T-bil and VWI grade, and **f** no correlation between BMI and VWI grade by Dr.N. *Hb* hemoglobin, *T-bil* total bilirubin, *BMI* body mass index, *VWI* Vein-weighted image

Several parameters, such as hemoglobin, total bilirubin, and BMI, can affect VWI grading. However, there were no correlations observed between hemoglobin (Dr.S; *rS* =  −0.092 *p* = 0.363, Dr.N; *rS* =  −0.029 *p* = 0.777) or BMI (Dr.S; *rS* =  −0.163 *p* = 0.134, Dr.N; *rS* =  −0.124 *p* = 0.256) and VWI grading by both doctors. Total bilirubin showed a weak positive correlation with VWI grading by both doctors (Dr.S; *rS* = 0.286 *p* = 0.004, Dr.N; *rS* = 0.262 *p* = 0.009) (Fig. 4). Fifteen patients had portosystemic shunts. These shunts did not affect the VWI grading by both doctors. (Dr.S; *rS* = 0.006 *p* = 0.954, Dr.N: *rS* =  −0.047 *p* = 0.643).

## Discussion

In this pilot study, we demonstrated that the vein-viewing application on iPhone could non-invasively detect AWV related to cirrhosis and GOV. This is the first report of the non-invasive method of simply taking AWV images that enables us to indicate the patients who should be applied for medical service and EGD.

Among the forty patients without GOV the mean platelet count was over 150,000/mm^3^, and mean liver stiffness was 17 kPa. This suggested that the cutoff levels for avoiding EGD in the Baveno VI guidelines were practical in indicating patients with a low risk of GOV.

In our study, age was an independent factor negatively related to GOV. This was an unexpected result. The difference in etiology may have caused this. The number of patients with alcoholism was larger in the participant group under 71 years of age compared to the older group (42% vs. 24%, p = 0.056). Moreover, most of the patients with alcoholism had cirrhosis (87.9%).

The image-based method for the prediction of GOV has been validated. Further development will enhance the usefulness of this approach in future medical diagnostics. Smartphones and mobile devices have rapidly become part of everyday life around the world. In the current situation with COVID-19 the role of on-line medical services is increasingly important. The vein-viewing application on the iPhone was not originally developed for medical purposes; however, we have established that it is useful in detecting AWV in cirrhotic patients in a medical context.

In this study, VWI-AWV grade 2 was related to the presence of cirrhosis, high Child–Pugh score, the presence of ascites, the presence of GOV, the presence of PHG, and low platelet count. A weak positive correlation between total bilirubin and VWI grading can also be associated with liver dysfunction. Furthermore, multivariate analysis demonstrated that VWI-AWV grade 2 was an independent factor related to non-treated GOV. GOV treatment would alter the hemodynamics, including AWV. Our approach is therefore more meaningful for diagnosing naïve than treated patients. Intriguingly, eight patients (22.9%) who were identified as grade 2 had no AWV when assessed by UI. Five patients with GOV (three were untreated) were included in the eight patients. The interrater reliability was lower in VWI-AWV grade 0–1, indicating that identifying a slight AWV was difficult. However, the identification of grade 2 AWV was significantly higher by VWI in both doctors, and the reliability of VWI-AWV grade 2 was satisfactory.

Among twenty-two VWI-AWV grade 2 patients who had no history of GOV treatment, six patients did not have any GOV. In this group, four patients (67%) had cirrhosis. VWI-AWV grade 2 may therefore have the potential to identify not only GOV but also cirrhosis. However, the other two patients had no cirrhosis and GOV; this would be an entirely false positive. Novel technology is warranted for the improvement of the vein-viewing application to minimize this outcome.

The role of artificial intelligence is also rapidly growing in the medical field, such as pathology, EGD, mammography, brain diseases, and COVID-19 diagnosis [24–28]. Deep learning of AWV structures would provide a highly reproducible diagnosis of AWV. It also means that each person can check themselves with such applications on mobile devices in the future. Our effort should be focused on quantifying the imaging capabilities of mobile devices on the human body and provide meaning and context to them.

This study has several limitations. The cohort studied represented a small group of patients on which EGD could be performed. Selection bias was therefore inevitable. However, based on the promising results of this pilot study, a large-scale cohort study will be conducted for validation. Presently, there are no available objective data on detectability differences for skin color. One user from Zimbabwe commented on the App Store review that the app was helpful to patients with dark skin. Although it may work for different skin colors, verification is warranted.

In summary, the vein-viewing application could non-invasively detect AWV related to the presence of cirrhosis and GOV. VWI-AWV grade 2 was an independent factor related to the presence of non-treated GOV. This result suggests a future direction of medicine using consumer mobile devices as medical devices. The camera lens will be like the eyes on "Baymax," a prototype healthcare-providing robot on Disney animation.

## Data Availability

The data that support the findings of this study are available from the corresponding author, Sugihara T., upon reasonable request.

## Abbreviations

AIH: Autoimmune hepatitis
APRI: Aspartate aminotransferase to platelet ratio
AST: Aspartate aminotransferase
ALT: Alanine aminotransferase
AWI: Abdominal wall varices
B-RTO: Balloon retrograde transvenous obliteration
EGD: Esophagogastroduodenoscopy
EVL: Endoscopic variceal ligation
EIS: Endoscopic injection sclerotherapy
FIB-4: Fibrosis index based on the four factors
GOV: Gastroesophageal varices
GR: Gastrorenal shunt
HBV: Hepatitis B virus
HCV: Hepatitis C virus
IMC: Inferior mesenteric caval shunt
MRI: Magnetic resonance of imaging
PBC: Primary biliary cholangitis
PHG: Portal hypertensive gastropathy
PT: Percent prothrombin time
RC: Red color sign
SR: Splenorenal shunt
UI: Unmanipulated image
VWI: Vein-weighed image
